# Detection of HPV DNA in vaginal samples self-collected by women living with HIV treated through the Brazilian public health system: Prevalence and analysis of risk factors

**DOI:** 10.1590/0037-8682-0277-2023

**Published:** 2023-10-09

**Authors:** Mariângela Freitas da Silveira, Romina Buffarini, Pâmela Cristina Gaspar, Hanalydia de Melo Machado, Maria Luiza Bazzo, Aline Scherer, Álvaro Luis Colusso, Ana Gabriela Álvares Travassos, Denise Arakaki-Sanchez, Djane Clarys Baia-da-Silva, Eduardo Campos de Oliveira, Isabella Ribeiro Zago, Marcos de Assis Moura, Marcus Vinicius Guimarães de Lacerda, Simone Murta Martins, Tânia Reuter, Valdir Monteiro Pinto, Waltesia Perini, Gerson Fernando Mendes Pereira, Angélica Espinosa Miranda

**Affiliations:** 1 Universidade Federal de Pelotas, Programa de Pós-graduação em Epidemiologia, Pelotas, RS, Brasil.; 2 Universidade Federal de Pelotas, Departamento de Medicina Social, Programa de Pós-Graduação em Epidemiologia, Pelotas, RS, Brasil.; 3 Ministério da Saúde, Departamento de HIV/aids, tuberculose, hepatites virais e infecções sexualmente transmissíveis, Brasília, DF, Brasil.; 4 Universidade de Brasília, Programa de Pós-Graduação em Saúde Coletiva, Brasília, DF, Brasil.; 5 Universidade Federal de Santa Catarina, Programa de Pós-Graduação em Farmácia, Laboratório de Biologia Molecular, Microbiologia e Sorologia, Florianópolis, SC, Brasil.; 6 Universidade Federal de Pelotas, Hospital Escola, Empresa Brasileira de Serviços Hospitalares, Pelotas, RS, Brasil.; 7 Secretaria do Estado de Saúde do Distrito Federal, Centro Especializado em Doenças Infecciosas do Distrito Federal, Brasília, DF, Brasil.; 8 Centro Especializado em Diagnóstico, Assistência e Pesquisa, Salvador, BA, Brasil.; 9 Fundação de Medicina Tropical Doutor Heitor Vieira Dourado, Manaus, AM, Brasil.; 10 Hospital Regional de São José Dr. Homero de Miranda Gomes, São José, SC, Brasil.; 11 Faculdade de Ciências Médicas e da Saúde de Juiz de Fora, Juiz de Fora, MG, Brasil.; 12 Universidade Federal de Juiz de Fora, Juiz de Fora, MG, Brasil.; 13 Universidade Federal do Espírito Santo, Hospital Universitário Cassiano Antônio Moraes, Vitória, ES, Brasil.; 14 Centro de Referência e Treinamento-DST/AIDS de São Paulo, São Paulo, SP, Brasil.; 15 Universidade Federal do Espírito Santo, Programa de Pós-Graduação em Doenças Infecciosas, Vitória, ES, Brasil.

**Keywords:** HPV, Screening, HIV, Uterine cervical neoplasms, Cervical intraepithelial neoplasia, HPV-DNA

## Abstract

**Background:**

Women living with human immunodeficiency virus (HIV) (WLWH) are more likely to be infected with the oncogenic human papillomavirus (HPV). We assessed the prevalence of high-risk (HR) (16/18/31/33/35/39/45/51/52/56/58/59/68/73/82), probable high-risk (pHR) (26/53/66), and low-risk (LR) (6/11/40/42/43/44/54/61/70) HPV types and their associated risk factors.

**Methods:**

This cross-sectional study of WLWH aged 18-64 years included one laboratory and eight HIV-specialty healthcare facilities in the pilot network. Descriptive statistics were used to assess sociodemographic and behavioral characteristics. Adjusted analyses were conducted to evaluate risk factors associated with HR and/or pHR HPV infection in WLWH.

**Results:**

From May/2021 to May/2022, 1,914 (92.5%) WLWH participated in the pilot study and had valid HPV-DNA results of self-collected vaginal samples. The median age of the participants was 45 years, 60.1% had ≥ 9 years of schooling, 80.5% were ≤ 18 years at first sexual intercourse, and 51.7% had > 4 sexual partners throughout life. The prevalence of any HPV type, HR HPV, pHR HPV, and LR HPV was 65.8%, 49.6%, 16.7%, and 40.0%, respectively. Age was inversely associated with pHR and/or HR-HPV (p < 0.001), and education level was inversely associated with HR-HPV (p = 0.003) types. Any HR or pHR was associated with being single (p = 0.029) and exchanging sex for drugs (p = 0.037).

**Conclusions:**

The prevalence of HPV, especially HR HPV, among WLWH is high in Brazil, highlighting the need for HPV screening in this population. Self-collection of vaginal samples is an important strategy for increasing testing access.

## INTRODUCTION

Human papillomavirus (HPV) belongs to the *Papillomaviridae* family and contains non-enveloped particles and double-stranded DNA genomes^1^. HPV is primarily transmitted through sexual contact. It is the most common sexually transmitted infection (STI), with higher transmissibility than that of herpes simplex virus 2 and HIV infections^2^. In Brazil, the POP-Brazil study, which included 6,387 patients from 26 Brazilian capitals and the Federal District of Brasilia, with a mean age of 21.6 years, found an HPV prevalence of 53.6%^3^.

Women living with HIV (WLWH) have a higher frequency of multiple infections, anogenital warts, intraepithelial lesions, and anogenital cancers resulting from HPV infection^4^. Particularly important in the origin of neoplasms are the high-risk HPV types (16, 18, 31, 33, 35, 39, 45, 51, 52, 56, 58, 59, 68, 73, and 82) and the probable high-risk HPV types (26, 53, and 66)^5^. A multicenter study of WLWH in Brazil found a prevalence of 28.4% for high-risk HPV, in addition to an association with abnormal cervical cytology, age < 35 years, and illicit drug use^6^. A study conducted in southern Brazil found HPV DNA in 76.4% of a sample of WLWH and a higher prevalence of infection with oncogenic HPV genotypes in the group of people living with HIV (65.2%) than in the HIV-seronegative group (28.6%) (p = 0.006), suggesting that WLWH are more likely to be infected with oncogenic HPV genotypes^7^. These findings highlight the importance of screening programs and specific diagnostic tests for this group.

HPV surveillance in WLWH is important because cervical cancer is considered an opportunistic disease in this group, as these women are more likely to develop preinvasive lesions that, if left untreated, can rapidly progress to invasive cancer^8^. WLWH have a 4- to 5-fold increased likelihood of developing invasive cervical cancer compared to women without HIV^8^. Strategies are needed to reduce infrastructure failures and access inequalities in order to successfully implement a screening program, such as self-sampling for HPV DNA testing, which has been proposed as an alternative method for cervical cancer screening. Self-sampling for HPV testing also has high sensitivity for detecting precancerous and cancerous lesions, with testing on self-samples being as accurate as on clinician samples^9^.

Cytology has been the primary screening tool for cervical cancer since its introduction into the Brazilian screening program in the 1970s^10^. A potential new strategy is to provide access to molecular biology assays for HPV testing by offering self-sampling kits through the Brazilian public health system for cervical cancer screening at the WLWH. In this study, we aimed to determine the prevalence of genital HPV infection in WLWH by using self-sampling devices, describe the most prevalent HPV types, and investigate the factors associated with infection with high-risk HPV, probable high-risk HPV, or both, in this group of women. 

## METHODS

We conducted a cross-sectional study of WLWH aged 18-64 years who had already had sexual intercourse across all five geographic regions of Brazil, from May 2021 to May 2022. The sample size was calculated by estimating that the proportion of women who underwent molecular HPV testing but did not undergo an up-to-date Papanicolaou (Pap) test would be 50%, requiring a sample size of 278 women per study site. Eight reference services for HIV/AIDS were included in the following cities: Pelotas and Florianópolis (southern region); São Paulo, Vitória, and Juiz de Fora (southeastern region); Brasília (midwestern region); Salvador (northeastern region); and Manaus (northern region). All WLWH aged between 18 and 64 years attended these services, and those who had already started sexual activity were invited to participate. The exclusion criteria were pregnancy, previous hysterectomy, and gynecological cancer. 

The study was approved by the Research Ethics Committees of the following participating centers: Universidade Federal de Pelotas (UFPel) Medical School, approval number 4,567,941 (CAAE: 43223521.7.1001.5317); STD/AIDS Reference and Training Center, approval number 4,692,020 (CAAE: 3223521.7.2002.5375); Cassiano Antônio de Moraes University Hospital affiliated with Universidade Federal do Espírito Santo (HUCAM/UFES), approval number 4,685,258 (CAAE: 43223521.7.2004.5071); Universidade Federal de Santa Catarina (UFSC), approval number 4,704,088 (CAAE: 43223521.7.2001.0121); Juiz de Fora School of Medical and Health Sciences, approval number 4,718,416 (CAAE: 43223521.7.2009.5103); “Doutor Heitor Vieira Dourado” Tropical Medicine Foundation, approval number 4,768,641 (CAAE: 43223521.7.2005.0005); Bahia State Department of Health (SESAB), approval number 4,829,852 (CAAE: 43223521.7.2003.0052); and Health Science Teaching and Research Foundation (FEPECS/SES/DF), approval number 5,000,655 (CAAE: 43223521.7.2006.5553). While attending the healthcare facilities, eligible women were informed of the study objectives and invited to participate. Those who agreed to participate provided written informed consent.

All women who agreed to participate answered an interview in which they provided sociodemographic and behavioral data (sexual activity, drug use, alcohol use, and tobacco use), received information on the method of self-collection of vaginal samples, and were asked about their health status. The women were also instructed on how to self-sample for molecular HPV testing according to the manufacturer’s instructions. 

Each study participant received a self-sampling kit and an information leaflet, which was read together with a trained health professional, who also answered any questions the participant might have had about the procedure. The participants individually self-collected vaginal samples in a private bathroom or room, without supervision, and delivered their samples to a health professional. Health professionals identified the samples by applying a pre-established coding scheme (anonymous codes). If any of the women had intercourse, vaginal cream, vaginal medication, or a vaginal or anal intimate shower in the last 48 h, it was registered. All samples were stored in accordance with the manufacturer’s instructions until they were sent to the laboratory for molecular HPV testing. 

DNA was extracted from all samples using the ReliaPrep™ Blood gDNA Miniprep System (Promega, USA) according to the manufacturer’s instructions. The Anyplex™ II HPV28 detection assay (Seegene, Seoul, Korea) was used for the qualitative detection of HPV DNA, discriminating the results into the following genotypes: 16, 18, 26, 31, 33, 35, 39, 45, 51, 52, 53, 56, 58, 59, 66, 68, 69, 73, 82, 6, 11, 40, 42, 43, 44, 54, 61, and 70. Dual priming oligonucleotide (DPO)-based real-time polymerase chain reaction (PCR) (DPO™, Seegene) detects all 28 HPV genotypes using the tagging oligonucleotide cleavage and extension (TOCE) technology with melting curve analysis (TOCE™, Seegene). This technology allows detection of multiple targets in a single real-time PCR fluorescence channel. According to the manufacturer’s instructions, 5 μL of DNA was added to each of the two wells containing 15 μL of reaction mixture (primer sets A and B). Real-time PCR amplification was performed in a CFX96 thermocycler (Bio-Rad, Hercules, CA, USA), and HPV L1 and human beta-globin genes were used as internal controls to monitor the efficiency of DNA purification and PCR inhibition. The test results were sent to the primary care provider at each site for follow-up. In the case of HPV-positive results, a follow-up visit with a clinician was scheduled for guidance and treatment according to the guidelines of the Brazilian Association for the Lower Genital Tract Pathology and Colposcopy^10^. The treatment was offered free of charge to all the participants. 

Statistical analyses included the overall prevalence of HPV, low-risk HPV, probable high-risk HPV, high-risk HPV, and high-risk and/or probable high-risk HPV infections in the study population. HPV types were classified: low-risk (types 6, 11, 40, 42, 43, 44, 54, 61, and 70), probable high-risk (types 26, 53, and 66), and high-risk (types 16, 18, 31, 33, 35, 39, 45, 51, 52, 56, 58, 59, 68, 73, and 82). Additionally, factors associated with HPV infection have been described, such as socioeconomic status, age, race/skin color, age at onset of sexual activity, and lifetime number of sex partners. The statistical analyses were performed using STATA, version 16.1 (StataCorp). Comparisons between women included in the study and those who refused to participate in terms of skin color and age were tested formally using the chi-square test in the case of proportions of skin color and the t-test to compare means of age. 

The following independent variables were analyzed: age, in complete years, categorized into four groups (18-29, 30-39, 40-49, and 50-73 years); education level, in years of schooling, categorized into four groups (0-4, 5-8, 9-11, and ≥ 12 years); self-reported race/skin color, collected according to the five categories of the Brazilian Institute of Geography and Statistics (white, yellow [Asian descent], red [native Brazilian], brown [mixed-race], and black)-due to the small number of women who self-reported as yellow (Asian descent) and red (native Brazilian), these two categories were not considered in bivariate analyses; marital status, collected and analyzed into four groups (single, separated/divorced, widowed, and lives with husband/partner); age at first sexual intercourse, categorized into four groups ( < 15, 15-18, 19-24, and ≥ 25); lifetime number of sex partners, categorized into four groups (1, 2-4, 5-10, and > 10); and parity, categorized into four groups (0, 1-2, 3-4, and ≥ 5). Exchange of sex for drugs or money over the past 12 months was used as a dichotomous variable. Smoking status was divided into three categories: never smoked, former smoker, and current smoker. Data on alcohol consumption in the previous month were classified into four categories: never, less than once a week, at least once a week, and every day.

Three primary outcomes were assessed: (a) infection with high-risk HPV, (b) infection with probable high-risk HPV, and (c) infection with high-risk HPV and/or probable high-risk HPV. For each outcome, the denominator was the total number of tests with valid results (n = 1,914) and the numerator was the presence of at least one HPV type in each category (high-risk, probable high-risk, high-risk and/or probable high-risk HPV). 

Poisson regression with robust variance was used for the adjusted analysis of factors associated with HPV infection in WLWH^11^ separately for all three outcomes. A 3-level hierarchical model was developed for the analysis. Level 1 (most distal): age, education level, and race/skin color. Level 2: Marital status, age at first sexual intercourse, number of lifetime sex partners, and parity. Level 3: Exchange of sex for drugs or money in the past 12 months, smoking, and alcohol consumption in the last month. For each outcome, the coefficients of each level are adjusted by the variables of the level itself and the variables of the previous level. Thus, the variables of level 3 (more proximal) are adjusted by the other variables of the same level and by the variables of levels 2 and 1.

## RESULTS

Of the eight healthcare facilities included in the study, 2,069 WLWH were invited to participate, 1,919 of whom agreed (92.8%) and 150 refused (7.2%) to participate. The women who refused to participate had a median age of 46.9 years (mean 45.5 years); 45.0% self-reported as White, 27.5% as Brown (mixed-race), and 27.5% as Black. Refusals were more common among white and brown women than among black women (p < 0.01); however, there was no difference in terms of mean age (p = 0.4). The number of self-collected vaginal samples per site ranged from 86 (Florianópolis) to 295 (Manaus) with a mean of 240 samples per site. The total number of vaginal samples self-collected by WLWH was 1,919, but five samples (0.3%) were considered invalid by the laboratory, for a total of 1,914 samples (99.7%) analyzed for detection of HPV DNA. 

The median age of the 1,914 WLWH participating in the study was 45 years (mean 44.7 years). Regarding race/skin color, 31.1% self-reported being white, 42.5% as brown (mixed-race), 24.6% as black, and 1.8% as yellow (Asian descent) or red (native Brazilian). Most participants were ≥ 40 years (68.8%), had 9 to 11 years of schooling (41.4%), and self-reported living with their husbands or partners (47.1%). More than half of the participants (57.5%) were 15 to 18 years of age at their first sexual intercourse and had one or two children (56.6%); 51.7% had had more than four sex partners throughout life. Only 14 participants (0.7%) reported exchanging sex for drugs, and 32 (1.7%) for money in the past 12 months (Table 1). 

**TABLE 1:** Sociodemographic and behavioral characteristics of WLWH in Brazil, 2021-2022 (n = 1,914).

Variable	n (%)
**Age (years)**	
18-29	199 (10.4)
30-39	398 (20.8)
40-49	641 (33.4)
50-73	680 (35.4)
**Education level (years of schooling)**	
0-4	278 (14.5)
5-8	487 (25.4)
9-11	795 (41.4)
12 or more	359 (18.7)
Race/skin color*	
White	594 (31.1)
Yellow (Asian descent)	26 (1.4)
Red (native Brazilian)	9 (0.5)
Brown (mixed-race)	811 (42.5)
Black	469 (24.6)
**Marital status**	
Single	571 (29.8)
Separated/divorced	288 (15.0)
Widowed	156 (8.1)
Lives with husband/partner	904 (47.1)
**Age at first sexual intercourse (years)**	
< 15	436 (23.0)
15-18	1093 (57.5)
19-24	332 (17.5)
25 or more	39 (2.0)
**Lifetime number of sex partners**	
1	122 (6.7)
2-4	759 (41.7)
5-10	642 (35.3)
> 10	298 (16.4)
**Parity**	
0	85 (4.9)
1-2	976 (56.6)
3-4	513 (29.7)
5 or more	151 (8.8)
**Exchange of sex for drugs - past 12 months**	
No	1898 (99.3)
Yes	14 (0.7)
**Exchange of sex for money - past 12 months**	
No	1878 (98.3)
Yes	32 (1.7)
**Smoking**	
Never smoked	1077 (56.4)
Former smoker	462 (24.2)
Current smoker	370 (19.4)
**Alcohol consumption - last month**	
Never	1023 (55.3)
< 1 time per week	435 (23.5)
At least 1 time per week	365 (19.7)
Every day	28 (1.5)
**City**	
Brasília	273 (14.2)
Florianópolis	86 (4.5)
Juiz de Fora	201 (10.5)
Manaus	295 (15.4)
Pelotas	280 (14.6)
Salvador	293 (15.3)
Sao Paulo	284 (14.8)
Vitória	207 (10.8)

Higher percentage of missing data for the variable lifetime number of sex partners (5.1%), followed by alcohol consumption (2.5%), and race/skin color (2.4%). **WLWH:** Women living with HIV. *Collected according to the five categories of the Brazilian Institute of Geography and Statistics.

Out of the 1,914 women living with HIV who were interviewed, 579 (30.2%) had no information on vaccination status, and only 9.9% reported having been immunized against HPV. Taking into account STI infections in the past 12 months, 7.6% (n = 146) of the participants reported being diagnosed with an STI by a physician. Additionally, 26 (1.4%) women indicated that they were unsure whether they had ever been diagnosed with an STI. The most frequently reported STIs were syphilis, herpes, and genital warts.

Of 1,914 WLWH who had valid test results, 49.6% had high-risk HPV (95% confidence interval [CI]: 47.4-51.9), 16.7% had probable high-risk HPV (95% CI: 15.1-18;5), and 40.0% had low-risk HPV (95% CI: 37.8-42.2). The prevalence of high-risk and/or probable high-risk HPV was 53.8% (95% CI: 51.6-56.0), whereas the prevalence of any HPV type (high-risk, probable high-risk, or low-risk) was 65.8% (95% CI: 63.7-67.9).

At least one high-risk HPV or probable high-risk HPV type was detected in the samples of the 1,269 women who tested positive for HPV (66.13% [95% CI: 64.0-68.2]). Of these, 74.8% (95% CI: 72.3-77.1) had at least one high-risk HPV type and 25% (95% CI: 22.9-27.7) had at least one probable high-risk HPV type. 

Table 2 shows the distribution of HPV types according to interview site, with the prevalence of high-risk HPV ranging from 34.5% (95% CI: 29.2-40.2) in São Paulo to 61.2% (95% CI: 55.5-66.6) in Manaus. 

**TABLE 2:** Distribution of HPV types among WLWH included in the study, by site of interview (n = 1,914).

City	High-risk HPV	Probable high-risk HPV	Low-risk HPV	High-risk and/or probable high-risk HPV	Total HPV*
	% (95% CI)	% (95% CI)	% (95% CI)	% (95% CI)	% (95% CI)
Brasília	44.5 (38.7-50.4)	14.0 (10.3-18.6)	35.3 (29.8-41.2)	47.8 (41.9-53.7)	63.6 (57.7-69.1)
Florianópolis	37.2 (27.7-47.9)	12.8 (7.2-21.6)	38.4 (28.7-49.0)	44.2 (34.1-54.8)	58.1 (47.5-68.1)
Juiz de Fora	50.5 (43.6-57.4)	25.0 (19.5-31.5)	50.0 (43.1-56.9)	59.0 (52.0-65.6)	73.0 (66.4-78.7)
Manaus	61.2 (55.5-66.6)	24.5 (19.9-29.7)	47.3 (41.6-53.0)	67.3 (61.8-72.5)	79.9 (75.0-84.1)
Pelotas	57.2 (51.3-62.9)	18.7 (14.5-23.7)	46.0 (40.3-51.9)	60.1 (54.2-65.7)	73.0 (67.5-77.9)
Salvador	46.8 (41.1-52.5)	16.7 (12.9-21.4)	39.2 (33.8-45.0)	50.5 (44.8-56.2)	61.8 (56.1-67.2)
Sao Paulo	34.5 (29.2-40.2)	8.8 (6.0-12.7)	22.2 (17.7-27.4)	37.0 (31.6-42.7)	45.1 (39.4-50.1)
Vitória	58.9 (52.1-65.4)	11.1 (7.5-16.2)	44.0 (37.3-50.8)	60.9 (54.1-67.3)	69.6 (63.0-75.5)
Total	49.6 (47.4-51.9)	16.7 (15.1-18.5)	40.0 (37.8-42.2)	53.8 (51.6-56.0)	65.8 (63.7-67.9)

*Total HPV: group consisting of high-risk, probable high-risk, and low-risk HPV types. **WLWH:** Women living with HIV. %: Prevalence; **95% CI:** 95% confidence interval.

**FIGURE 1:**
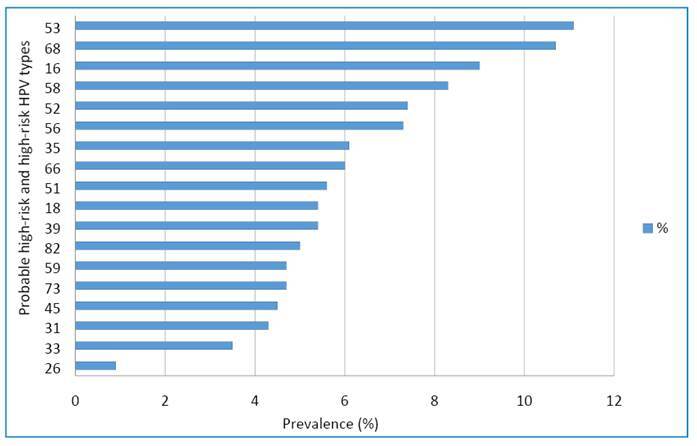
Probable high-risk and high-risk HPV types found in women with valid HPV DNA testing (n = 1,914).

Figure 1 shows the distribution of probable high- and high-risk HPV types in samples that were positive for at least one HPV type. The most common type was HPV-53 (11.1%), followed by HPV-68 (10.7%) and HPV-16 (9.0%). 

The factors associated with probable high-risk and/or high-risk HPV infection in the univariate analyses were age, education level, lifetime number of sexual partners, exchange of sex for money (past 12 months), and alcohol consumption (last month) (Table 3). Regarding the adjusted analysis of possible factors associated revealed that age was inversely associated with the presence of high-risk HPV, probable high-risk HPV, and both HPV types - the highest prevalence of high-risk HPV was found in women aged 18-29 years (61.3% [95% CI: 54.4-67.8]) and the lowest prevalence in women aged 50-73 years (45.7% [95% CI: 42.0-49.5]). Education level was also inversely associated with high-risk HPV (p = 0.003) types. The presence of any type of high-risk HPV was associated with marital status and was more common in single women (p = 0.029). Exchange of sex for drugs (in the past 12 months) was associated with probable high-risk HPV detection (p = 0.037). There was no association between race/skin color, age at first sexual intercourse, lifetime number of sexual partners, parity, exchange of sex for money, smoking, or alcohol consumption (Table 4). 

**TABLE 3:** Prevalence of different HPV types in women with valid tests according to sociodemographic and behavioral characteristics (n = 1914).

Variable	High-risk HPV	Probable high-risk HPV	Total HPV*
	% (95% CI)	% (95% CI)	% (95% CI)
**Age (years)**	p < 0.001	p < 0.001	p < 0.001
18-29	61.3 (54.4-67.8)	25.1 (19.6-31.6)	65.3 (58.5-71.6)
30-39	55.3 (50.4-60.1)	20.1 (16.4-24.3)	60.6 (55.7-65.2)
40-49	46.6 (42.7-50.4)	15.5 (12.9-18.5)	50.3 (46.4-54.2)
50-73	45.7 (42.0-49.5)	13.4 (11.1-16.2)	49.7 (45.9-53.5)
**Education level (years of schooling)**	p = 0.005	p = 0.29	p = 0.002
0-4	54.3 (48.4-60.1)	19.6 (15.3-24.7)	59.1 (53.2-64.7)
5-8	54.0 (49.6-58.4)	17.7 (14.6-21.4)	58.1 (53.7-62.5)
9-11	48.2 (44.8-51.7)	16.2 (13.8-19.0)	52.5 (49.0-56.0)
≥ 12	43.2 (38.1-48.4)	14.2 (11.0-18.2)	46.8 (41.7-52.0)
**Race/skin color**	p = 0.532	p = 0.38	p = 0.599
White	48.2 (44.2-52.3)	15.1 (12.4-18.2)	52.5 (48.4-56.5)
Brown (mixed-race)	51.2 (47.8-54.7)	17.4 (14.9-20.2)	55.2 (51.7-58.6)
Black	49.6 (45.1-54.1)	17.9 (14.7-21.7)	54.3 (49.7-58.7)
**Marital status**	p = 0.114	p = 0.03	p = 0.045
Single	52.1 (48.0-56.2)	20.2 (17.1-23.7)	57.2 (53.1-61.2)
Widowed/separated/divorced	51.6 (46.9-56.2)	14.9 (11.9-18.6)	55.4 (50.8-60.0)
Lives with husband/partner	47.1 (43.9-50.4)	15.4 (13.2-17.9)	50.9 (47.6-54.1)
**Age at first sexual intercourse (years)**	p = 0.416	p = 0.113	p = 0.187
< 15	52.2 (47.5-56.8)	19.8 (16.3-23.8)	56.6 (51.8-61.1)
15-18	49.7 (46.7-52.6)	16.3 (14.3-18.7)	54.4 (51.4-57.3)
19-24	46.1 (40.8-51.5)	13.6 (10.3-17.7)	48.8 (43.4-54.2)
≥ 25	51.3 (36.0-66.4)	20.5 (10.6-36.0)	53.8 (38.3-68.7)
**Lifetime number of sex partners**	p = 0.026	p = 0.462	p = 0.045
1	44.6 (36.0-53.6)	16.5 (10.9-24.2)	47.1 (38.4-56.0)
2-4	48.6 (45.1-52.2)	14.8 (12.4-17.5)	53.5 (49.9-57.0)
5-10	53.0 (49.2-56.9)	17.9 (15.2-21.1)	56.5 (52.6-60.3)
> 10	43.1 (37.6-48.8)	16.8 (13.0-21.5)	47.8 (42.2-53.5)
**Parity**	p = 0.079	p = 0.148	p = 0.073
0	47.1 (36.7-57.7)	17.6 (10.9-27.2)	52.9 (42.3-63.3)
1-2	47.4 (44.3-50.6)	15.4 (13.3-17.8)	51.4 (48.3-54.6)
3-4	52.7 (48.4-57.0)	16.0 (13.1-19.5)	57.0 (52.7-61.3)
≥ 5	56.4 (48.3-64.1)	22.8 (16.8-30.2)	60.4 (52.3-67.9)
**Exchange of sex for drugs - past 12 months**	p = 0.296	p = 0.07	p = 0.282
No	49.4 (47.2-51.7)	16.6 (15.0-18.3)	53.6 (51.4-55.9)
Yes	64.3 (37.6-84.3)	35.7 (15.7-62.4)	71.4 (43.9-88.9)
**Exchange of sex for money - past 12 months**	p = 0.032	p = 0.093	p = 0.107
No	49.2 (46.9-51.4)	16.6 (14.9-18.3)	53.4 (51.2-55.7)
Yes	68.8 (51.0-82.3)	28.1 (15.3-45.8)	68.8 (51.0-82.3)
**Smoking**	p = 0.392	p = 0.963	p = 0.374
Never smoked	48.3 (45.3-51.3)	16.4 (14.4-18.8)	52.7 (49.7-55.7)
Former smoker	50.3 (45.8-54.9)	17.0 (13.8-20.7)	53.6 (49.0-58.1)
Current smoker	52.3 (47.2-57.4)	16.8 (13.3-21.0)	56.9 (51.8-61.9)
**Alcohol consumption - last month**	p = 0.009	p = 0.003	p = 0.005
Never	46.9 (43.8-49.9)	14.1 (12.1-16.4)	50.8 (47.7-53.8)
< 1 time per week	47.1 (42.5-51.8)	15.9 (12.7-19.6)	51.3 (46.6-55.9)
At least 1 time per week	56.9 (51.7-61.9)	22.3 (18.3-26.8)	61.3 (56.2-66.1)
Every day	50.0 (32.3-67.7)	21.4 (10.0-40.2)	57.1 (38.7-73.8)

*Total HPV: group consisting of high-risk and/or probable high-risk. Yellow (Asian descent) and red (native Brazilian) race/skin color categories were not considered in this analysis because of insufficient N. The p-value corresponds to the chi-squared test for variables with three or more categories and Fisher’s exact test for dichotomous variables. **%:** Prevalence; **95% CI:** 95% confidence interval; p: p-value

**TABLE 4:** Adjusted analysis of different HPV types in women with valid tests according to sociodemographic and behavioral characteristics.

Variable	High-risk HPV	Probable high-risk HPV	Total HPV*
	PR (95% CI)	PR (95% CI)	PR (95% CI)
**Age (years)**	p < 0.001	p < 0.001	p < 0.001
18-29	1.4 (1.2-1.6)	1.9 (1.4-2.7)	1.3 (1.2-1.5)
30-39	1.2 (1.1-1.4)	1.5 (1.1-2.0)	1.2 (1.1-1.4)
40-49	1.0 (0.9-1.2)	1.2 (0.9-1.5)	1.0 (0.9-1.1)
50-73	Ref.	Ref.	Ref.
**Education level (years of schooling)**	p = 0.003	p = 0.161	p = 0.001
0-4	1.3 (1.1-1.6)	1.5 (1.0-2.1)	1.3 (1.1-1.5)
5-8	1.2 (1.1-1.4)	1.2 (0.9-1.6)	1.2 (1.1-1.4)
9-11	1.1 (1.0-1.3)	1.1 (0.8-1.5)	1.1 (1.0-1.3)
≥ 12	Ref.	Ref.	Ref.
**Race/skin color**	p = 0.606	p = 0.831	p = 0.739
White	Ref.	Ref.	Ref.
Brown (mixed-race)	1.0 (0.9-1.1)	1.1 (0.8-1.4)	1.0 (0.9-1.1)
Black	1.0 (0.9-1.1)	1.1 (0.8-1.4)	1.0 (0.9-1.1)
**Marital status**	p = 0.029	p = 0.101	p = 0.014
Single	1.1 (1.0-1.2)	1.3 (1.0-1.7)	1.1 (1.0-1.3)
Widowed/separated/divorced	1.2 (1.0-1.4)	1.2 (0.9-1.6)	1.2 (1.0-1.3)
Lives with husband/partner	Ref.	Ref.	Ref.
**Age at first sexual intercourse (years)**	p = 0.662	p = 0.521	p = 0.873
< 15	Ref.	Ref.	Ref.
15-18	1.0 (0.9-1.1)	1.0 (0.7-1.3)	1.0 (0.9-1.2)
19-24	1.1 (0.9-1.3)	0.9 (0.6-1.4)	1.0 (0.9-1.2)
≥ 25	1.2 (0.8-1.8)	1.7 (0.7-3.8)	1.1 (0.8-1.7)
**Lifetime number of sex partners**	p = 0.077	p = 0.275	p = 0.076
1	Ref.	Ref.	Ref.
2-4	1.1 (0.9-1.4)	0.9 (0.6-1.6)	1.1 (0.9-1.4)
5-10	1.2 (0.9-1.5)	1.2 (0.7-2.1)	1.2 (1.0-1.5)
> 10	1.0 (0.8-1.3)	1.0 (0.6-1.8)	1.0 (0.8-1.3)
**Parity**	p = 0.264	p = 0.506	p = 0.328
0	Ref.	Ref.	Ref.
1-2	1.0 (0.8-1.3)	0.9 (0.6-1.5)	1.0 (0.8-1.2)
3-4	1.1 (0.9-1.4)	1.0 (0.6-1.6)	1.1 (0.9-1.3)
≥ 5	1.1 (0.9-1.5)	1.2 (0.7-2.3)	1.1 (0.8-1.4)
**Exchange of sex for drugs - past 12 months**	p = 0.544	p = 0.037	p = 0.173
No	Ref.	Ref.	Ref.
Yes	1.2 (0.7-2.1)	2.5 (1.1-5.9)	1.3 (0.9-2.0)
**Exchange of sex for money - past 12 months**	p = 0.837	p = 0.769	p = 0.769
No	Ref.	Ref.	Ref.
Yes	1.0 (0.7-1.6)	1.1 (0.5-2.3)	0.9 (0.6-1.4)
**Smoking**	p = 0.545	p = 0.947	p = 0.39
Never smoked	Ref.	Ref.	Ref.
Former smoker	1.0 (0.9-1.1)	1.0 (0.8-1.4)	1.0 (0.9-1.1)
Current smoker	1.1 (0.9-1.2)	1.0 (0.8-1.4)	1.1 (1.0-1.2)
**Alcohol consumption - last month**	p = 0.122	p = 0.322	p = 0.125
Never	Ref.	Ref.	Ref.
< 1 time per week	1.0 (0.9-1.1)	1.1 (0.8-1.5)	1.0 (0.9-1.1)
At least 1 time per week	1.2 (1.0-1.3)	1.3 (1.0-1.8)	1.1 (1.0-1.3)
Every day	1.0 (0.6-1.5)	1.1 (0.4-2.8)	1.0 (0.7-1.4)

*Total HPV: group consisting of high-risk and/or probable high-risk. Yellow (Asian descent) and red (native Brazilian) race/skin color categories were not considered in this analysis because of insufficient N. **PR:** Prevalence ratio; **95% CI:** 95% confidence interval; **p:** p-value. P-values correspond to the z-test from Poisson regression with robust variance.

## DISCUSSION

The HPV prevalence found in WLWH in the eight HIV specialty healthcare facilities distributed across eight Brazilian states was 49.6% for high-risk HPV and 16.7% for probable high-risk HPV. This prevalence was higher than that of a multicenter study conducted in Brazil, which reported a 28.4% prevalence of high-risk HPV in WLWH^6^, and lower than that reported in a study conducted in southern Brazil, which found a higher prevalence of infection with oncogenic HPV genotypes in the group of people living with HIV (65.2%) than in the HIV-seronegative group (28.6%), suggesting that WLWH are more likely to be infected with oncogenic HPV genotypes^7^. A systematic review found a similar prevalence of HPV-16 in WLWH and HIV-negative women, but a higher prevalence of HPV-58, HPV-31, and HPV-52 in WLWH and higher prevalence of HPV-18 in HIV-negative women^12^. Another systematic review of WLWH in Brazil reported a pooled prevalence of HPV infection of 62% (95% CI: 55-68; I^2^ = 96.98%; p < 0.001), with a prevalence of high-risk HPV of 40% (95% CI: 54-68; I^2^ = 94.23%; p < 0.001), of which the most reported types were HPV-16 (16%) and HPV-58 (6%)^13^. Studies show that approximately 85% of low-grade cervical lesions contain oncogenic HPV types and should be evaluated^14^ and that some individual characteristics are predisposing factors for the development of lesions, such as smoking, immunodeficiency diseases (including those caused by HIV infection), malnutrition, cancer, and the use of immunosuppressants^15^. 

In the present study, in which most participants were ≥ 40 years, more than half reported having their first sexual intercourse between 15 and 18 years of age, and a higher prevalence of high-risk HPV was observed in women aged 18-29 years. We observed an inverse association between the presence of high-risk HPV (p = 0.003) and low education levels, which is consistent with the findings of Monteiro et al. (2021)^16^ and Gaspar et al. (2015)^17^. Controversially, a Brazilian multicenter study did not identify any association with educational level. This study aggregated WLHV with either less or more than 4 years of education into two groups^6^, unlike our study, which divided the group into four categories based on years of education. We observed a positive association between being single and high-risk HPV (p = 0.029); however, this was a small effect, probably due to the limited sample size. While Miranda et al. (2017)^6^ did not report an association, two studies conducted in Brazil^16^ ^,^ ^18^ and one conducted in the United States^19^ also found a positive association between marital status and HPV infections, with higher HPV prevalence in single/unmarried women. A total of 32 (1.7%) participants reported exchanging sex for drugs in the past 12 months, and which was associated with probable high-risk HPV detection (p = 0.037). 

A limitation of this study is that some interview questions addressed intimate matters that relied on the recall of past events, which may have underestimated the risk factors for certain morbidities associated with sexual and reproductive health. However, the high participation rate and few lost samples highlight the importance of this study, with representation across all five geographic regions of the country, to produce evidence for the development of public policies for WLWH in Brazil. The large number of unanswered responses to the vaccination question made it impossible to analyze the presence of HPV infection and vaccination status. Information on CD4 count, viral load, and antiretroviral treatment was not collected. The questionnaire was summarized to reduce the interview and collection time for the WLWH, as we took advantage of any type of contact the woman had with the service. 

There is a growing awareness of the need to maximize synergies between the HIV/AIDS response and efforts to prevent, diagnose, and treat cervical cancer through HPV vaccination, education, screening, and treatment strategies. Similarly, existing HIV programs can play a strategic role in expanding cervical cancer prevention services^20^. The causal relationship between high-risk HPV infection and precancerous lesions or cervical cancer has led to the development of strategies to improve cervical cancer screening and prevention, which are highly relevant to public health^21^. This situation needs to be addressed along the care pathway as WLWH are living longer and healthier lives because of antiretroviral therapy for HIV infection, reason why it is important that they do not become ill from other comorbidities, including cervical cancer.

Several health systems have considered switching from cytology to HPV DNA testing for cervical cancer screening based on randomized trials that have demonstrated the superior effectiveness of the latter, with improved sensitivity for detecting lesions, that is, reducing the risk of false-negative results, and extended screening intervals when HPV is not detected^22^ ^-^ ^24^. Currently, cervical cancer screening is based on the results of cytological and/or HPV DNA testing using molecular biology assays^17^. Molecular high-risk HPV testing can detect more than 90% of cases of high-grade intraepithelial neoplasia and; therefore, has been proposed as an option for cervical screening, mainly in countries with a well-established infrastructure^25^ ^,^ ^26^. Given the association between high-risk HPV types and cervical cancer, the detection and differentiation of these types are clinically relevant. Therefore, tests that can detect and discriminate between these HPV types are preferred^14^ ^,^ ^23^ ^,^ ^27^. In addition to early detection of high-risk HPV, molecular testing is also related to improving the sensitivity of cytology for detecting malignant lesions when there is prior knowledge of the existence of HPV infection, as demonstrated by Martins et al. (2018), who observed an increase from 60.0% to 86.7%^28^.

This study used a self-sampling strategy to optimize the primary screening of WLWH during routine visits to an infectious disease service. Self-collection of vaginal samples for HPV testing has high sensitivity for detecting precancerous and cancerous lesions, with an accuracy similar to that of clinician-collected samples^9^ and appears to be a cost-effective way to increase screening coverage, especially in countries with difficulties in implementing the traditional method. The present results showed high acceptance among WLWH, few inappropriate samples, and a high prevalence of high-risk HPV in this population. Therefore, these data underscore the importance of including tests for the detection of HPV DNA through molecular biology assays in the screening program for WLWH, especially using a self-sampling strategy, to increase access to and coverage of cervical cancer prevention measures.
